# Clinically Relevant Properties of 3D Printable Materials for Intraoral Use in Orthodontics: A Critical Review of the Literature

**DOI:** 10.3390/ma16062166

**Published:** 2023-03-08

**Authors:** Cecilia Goracci, Jovana Juloski, Claudio D’Amico, Dario Balestra, Alessandra Volpe, Jelena Juloski, Alessandro Vichi

**Affiliations:** 1 Department of Medical Biotechnologies, University of Siena, 53100 Siena, Italy; 2 Department of Orthodontics, School of Dental Medicine, University of Belgrade, 11000 Belgrade, Serbia; 3 School of Dental Medicine, Alfonso X El Sabio University, 28691 Madrid, Spain; 4 Clinic for Paediatric and Preventive Dentistry, School of Dental Medicine, University of Belgrade, 11000 Belgrade, Serbia; 5 Dental Academy, University of Portsmouth, Portsmouth PO1 2QG, UK

**Keywords:** 3D printing, additive manufacturing, rapid prototyping, orthodontics, materials, review

## Abstract

The review aimed at analyzing the evidence available on 3D printable materials and techniques used for the fabrication of orthodontic appliances, focusing on materials properties that are clinically relevant. MEDLINE/PubMed, Scopus, and Cochrane Library databases were searched. Starting from an initial retrieval of 669 citations, 47 articles were finally included in the qualitative review. Several articles presented proof-of-concept clinical cases describing the digital workflow to manufacture a variety of appliances. Clinical studies other than these case reports are not available. The fabrication of aligners is the most investigated application of 3D printing in orthodontics, and, among materials, Dental LT Clear Resin (Formlabs) has been tested in several studies, although Tera Harz TC-85 (Graphy) is currently the only material specifically marketed for direct printing of aligners. Tests of the mechanical properties of aligners materials lacked homogeneity in the protocols, while biocompatibility tests failed to assess the influence of intraoral conditions on eluents release. The aesthetic properties of 3D-printed appliances are largely unexplored. The evidence on 3D-printed metallic appliances is also limited. The scientific evidence on 3D printable orthodontic materials and techniques should be strengthened by defining international standards for laboratory testing and by starting the necessary clinical trials.

## 1. Introduction

Technological advancements in three-dimensional (3D) imaging, digital modeling, and additive manufacturing have introduced significant changes in dentistry [1]. In particular, the application of 3D printing to the orthodontic workflow has enabled the digital production of dental models, aligners, retainers, nightguards, occlusal splints, indirect bonding trays, surgical guides for implant placement, and metal frameworks of various orthodontic appliances [2,3,4]. Such manufacturing can also be carried out ‘in-house’. In-house fabrication implies that from treatment planning to appliance delivery, every step is handled in the orthodontic office. In-house manufacturing allows to reduce the product delivery time, eliminates shipping costs, and decreases the waste material by avoiding models’ fabrication and thermoforming [1,5].

It has also been claimed that 3D printing provides greater opportunities for the customization of intraoral and extraoral devices [1,5,6,7,8].

Despite these reported advantages, the adoption of the digital workflow still challenges the practitioner with the burden of an initial investment in equipment and the need for adequate training [1]. Panayi and Eliades [9] have recently pointed out that the enthusiasm for the use of 3D printing should not conceal that scarce evidence has so far been published on key characteristics of 3D-printed devices, such as mechanical properties and biocompatibility. Particularly, the author mentioned that the effect of intraoral aging on the properties of 3D-printed materials is still largely unexplored [9]. Information is also lacking on the electromechanical properties of 3D-printed alloys, such as Co-Cr, that determine the resistance of the device to corrosion, ions release, and surface alterations [10]. Aside from stability in the oral environment, aesthetic characteristics are also key properties for orthodontic appliances and should be properly evaluated for 3D-printed devices [11].

It therefore appeared timely and relevant to explore the currently available scientific literature on the properties of 3D-printable materials that impact the clinical performance of orthodontic appliances. At this objective, the present critical review was directed.

## 2. Materials and Methods

The literature review was conducted based on the following question: ‘What is the scientific evidence currently available on 3D printable materials and techniques used for the fabrication of intraoral orthodontic appliances?’ The search was focused on the characteristics that are relevant to the clinical service of the appliance, such as fit, mechanical, biological, and aesthetic properties of the materials.

### 2.1. Inclusion and Exclusion Criteria

The review was restricted to the evidence on devices meant for prolonged intraoral use, excluding temporarily used devices. Thereby, studies on materials used to produce orthodontic casts, trays for indirect bonding, orthognathic surgery guides for jaws repositioning, and surgical guides for the positioning of temporary anchorage devices (TADs) were not included. In addition, editorials, communications, comments on previous articles, and conference excerpts were excluded. 

### 2.2. Information Sources and Research Strategies

An electronic search was conducted using the following databases: MEDLINE/PubMed, Scopus, Cochrane Library, with no restrictions on the publication date. The last database consultation was on 19 November 2022. Only publications in the English language were selected. Table 1 reports terms and search strategy for each database, and the number of documents retrieved.

### 2.3. Sources of Evidence Selection

Each article was evaluated through a three-step process, which consequently took into consideration the title, abstract, and full text of the manuscript. Two investigators (C.D., C.G.), working independently, judged whether each article met the inclusion criteria and was relevant to the objective of the review. In case of disagreement between the investigators, a shared decision was reached upon discussion.

## 3. Results

Querying the databases with the defined search terms initially retrieved 669 citations (MEDLINE/PubMed: *n* = 464; Scopus: *n* = 205; Cochrane Library: *n* = 0 reviews). After identifying and eliminating duplicates, the title screening process excluded articles not evaluating materials for 3D printing, as well as those investigating printing materials for casts, trays for indirect bonding, orthognathic surgical guides, and surgical guides for the insertion of TADs. The abstracts of 76 articles were then carefully analyzed; 21 of them were considered irrelevant to the objective of the review and were excluded. 

The full texts of the remaining 55 articles were read, and 44 articles were eventually included in the qualitative analysis. Among the references of the selected articles, three additional pertinent studies were identified and added to the qualitative analysis. 

The findings of the reviewed studies are reported in the following sections: 1. Case Reports and Proofs of Concept, 2. Mechanical and Geometric Properties, 3. Biocompatibility, and 4. Aesthetic Properties. 

A quantitative analysis of the collected evidence was not performed. 

**Figure 1:**
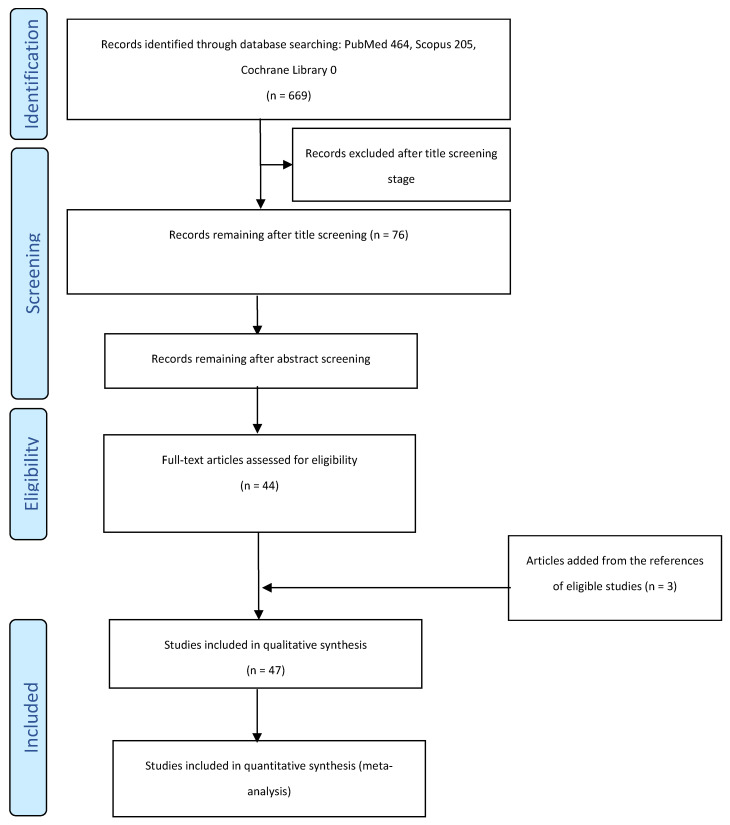
Study selection flow chart.

Figure 1 presents the study selection flowchart.

### 3.1. Case Reports and Proofs of Concept Clinical Cases

Several articles described the digital workflow for 3D-printed intraoral orthodontic devices. 

Two case reports were related to the production of 3D-printed band-and-loop space maintainers using a titanium-based powder metal material (Ti64 Gd23; LPW Technology Ltd., Cheshire, UK) by micro selective laser sintering (SLS) or a clear photopolymer resin (Form 2, Formlabs Inc. Somerville, MA, USA) by stereolithography [12,13]. Liang et al. [14] described the treatment of skeletal Class III malocclusion and mid-face deficiency with customized mini-plates for the anchorage of elastic tractions in the protraction of the maxilla. The mini-plates were designed according to maxillary bone anatomy and the position of the roots and then 3D printed by selective laser melting (SLM) of titanium powder [14]. Similarly, Kim et al. [8] used a customized bone-anchored maxillary protraction device, 3D printed in a titanium alloy with SLS, to hook intermaxillary elastics in the treatment of two patients with Class III malocclusion. Graf et al. [15] showed that a digital workflow involving intraoral digital scan, digital design, direct 3D metal printing via SLM (Concept Laser, Lichtenfels, Germany), and laser welding could be efficient for constructing Hyrax-type rapid palatal expanders (RPEs), also supported by mini-implants [16]. Cozzani et al. [17] described the digital production of a Haas-type RPE that was 3D printed in a cobalt–chromium alloy with an SLM printer (DWS D20, 3DRPD, Mouilleron-le-Captif, France). A series of cases were presented to demonstrate the versatility of 3D metal printing [7]. Hyrax-type RPEs with different designs, Herbst appliance, and lingual arch were printed in a cobalt-chromium alloy (Remanium Star, Dentaurum, Ispringen, Germany) using the MLab SLM device (Concept Laser, Lichtenfels, Germany) [7]. 

Van der Meer et al. [18] presented the digital manufacturing of a removable orthodontic appliance featuring a 3D-printed acrylic plate with clasps and springs bent by a robot. Another study showed a prototype of the Hawley retainer produced using only digital technology [19]. The resin base was built in ClearVue by an SLA 250/50 machine, while Adams clasps and a fitted labial bow were built in Co-Cr alloy SP2 (EOSINT M 270, Electro Optical Systems, Krailling, Germany), still using additive manufacturing (3D Systems) [19]. Nasef et al. [20] reported a procedure for printing a retainer with SLS of a polyamide powder (Fine Polyamide PA 2200, EOS GmbH, München, Germany). Fayyaz Ahamed et al. [21] and Thurzo et al. [22] described the possibility of 3D printing various customized orthodontic auxiliaries. Particularly, the Visijet FTX Clear SLA resin (3D System, Rock Hill, SC, USA) was used to produce retraction hooks and aligner attachments, while Visijet FTX Green SLA resin (3D System) was employed for lingual retainers, and ABS (MakerBot Industries, Brooklyn, NY, USA) for printing bite turbos by fused deposition modeling (FDM) [21]. Dental LT Clear Resin (Formlabs Inc.) was utilized to manufacture two different designs of power arms printed with SLA (Form 2, Formlabs Inc.) [22]. The new, improved design showed higher strength, lower stress, and less frequent debonding and cracking compared to the older design [22].

Graf et al. [23] utilized a biocompatible Class IIa acrylic resin (Ortho Clear, NextDent, Soesterberg, the Netherlands) for in-house printing of Twin Block appliances used for Class II malocclusion treatment in two patients. The appliances did not include any metallic clasp. The authors suggested that further research should be performed on the mechanical properties, color, and longevity of the material and that the success of clasp-free retention should be further assessed [23].

One article proposed a fully digital workflow to individually design brackets and 3D print them with digital light technology (DLP). Technical aspects, advantages, disadvantages, and future directions of this application were presented [24].

### 3.2. Mechanical and Geometric Properties 

#### 3.2.1. 3D-Printed Aligners and Retainers

A great interest surrounds the use of 3D printing technologies to produce dental aligners and retainers [25]. 

The accuracy of 3D-printed retainers and aligners has been evaluated with reference to printing technology [26], printing material [27,28,29], printing orientation [30,31,32], and post-printing protocols [30]. In addition, thickness [27], fit [33,34], mechanical, and thermo-mechanical properties of 3D-printed aligners and retainers have been investigated [28,29,30,35,36,37,38,39,40,41,42,43,44].

The precision, trueness, and accuracy of retainers printed with four different 3D printing technologies were compared [26]. Retainers were fabricated with an SLA printer (Form 3, Formlabs), DLP printer (MoonRay, SprintRay Inc., Los Angeles, CA, USA), continuous DLP (cDLP) printer (Envision One cDLM Dental, EnvisionTEC, Dearborn, MI, USA), and polyjet photopolymer (PPP) printer (Object Eden260VS, Stratasys, Eden Prarie, MN, USA). The manufacturers’ recommendations regarding material, print angulation, print layer heights, curing modalities, and post-printing procedures specific to each system were followed. The results of the study showed that there were statistically significant differences among the different 3D printing technologies. Specifically, PPP and SLA printers printed the most accurate retainers, while DLP and cDLP produced the most precise retainers [26].

Edelmann et al. [27] assessed the thickness of the digitally designed aligners as an indicator of digital manufacturing accuracy. The results showed that aligners printed in Dental LT Clear V1 or Gray V4 resin (Formlabs Inc.) with an SLA printer were thicker than the digital design. Additionally, aligners printed in Dental LT clear V1 showed greater variability in thickness than those printed in Gray V4 material [27]. In accordance with this finding, Lee et al. [29] reported that the thickness of 3D-printed aligners manufactured with a DLP printer (Uniz 4K, Uniz, San Diego, CA, USA) in the newly developed photocurable shape memory resin Tera Harz TC-85 (Graphy Inc., Seoul, Republic of Korea) was 12% greater than the value set in the digital design. The concern was raised that the increase in thickness compared to the digital design could affect the biomechanics and efficiency of the aligner. Jindal et al. [28] observed that aligners printed in biocompatible Dental LT resin (Formlabs Inc.) with SLA printer (Form 2, Formlabs Inc.) had greater geometrical accuracy than conventionally thermoformed aligners (Duran, Scheu-Dental GmbH, Iserlohn, Germany). 

The influence of printing orientation on the dimensional accuracy of the 3D-printed aligners was also assessed [30,31,32]. Mc Carty et al. reported that print orientation did not significantly affect the accuracy of aligners printed in biocompatible Dental LT resin (Formlabs Inc.), and only localized areas of dimensional deviations were detected [30]. The aligner’s accuracy was also unaffected by the duration of UV light exposure in the post-print process [30]. Still, for Dental LT Clear Resin (Formlabs Inc.), Williams et al. [31] found that all the investigated printing orientations showed clinically acceptable accuracy at the incisal edges and cup tips, while most of the accuracy errors were localized on the facial surfaces of anterior teeth. Printing angulation was indeed found to affect printing time and amount of consumed resin, with 15° being the most time-efficient setting and 45° the most cost-effective one [31]. Boyer et al. [32] found that printing orientation influenced the dimensional accuracy of Gray V4 resin (Formlabs Inc.). The most precise aligners were those printed in 90° orientation, i.e., with the aligner being built perpendicularly to the print platform, while in other orientations, the accuracy of the prints was rated as clinically unacceptable [32]. 

Cole et al. [33] compared the adaptation of retainers 3D printed in Dental LT Clear Resin (Formlabs Inc.), vacuum formed from Essix Plus sheets (Dentsply International, York, PA, USA), and thermoformed Vivera retainers (Align Technology, San Jose, CA, USA). Fit was evaluated as the distance between the retainer and the model at specific reference points by means of software. The 3D-printed retainers had the greatest deviation values, while the vacuum-formed ones showed the smallest discrepancies [33]. The deviation values of 3D-printed retainers were higher at flat surfaces, while the fit was better at incisal edges and cusp tips. Nevertheless, for all retainer types, the deviations were clinically acceptable [33].

Martorelli et al. [34] evaluated the adaptation of aligners printed with a photopolymer jet printer and compared it with that of aligners milled with a computer numerical control (CNC) milling machine. The CNC aligners were found to fit better and requested a shorter time for teeth straightening. However, it was not reported what materials were used with the different manufacturing technologies, and the adaptation was assessed only through a questionnaire addressed to a sample of six patients [34].

Koenig et al. [35] compared the dimensional accuracy of aligners produced using polyurethane thermoforming sheets (Zendura FLXTM, Zendura Dental, Fremont, CA, USA) and Tera Harz TC-85 (Graphy Inc.) 3D printed with the SprintRay Pro 3D device (SprintRay Inc.). It was reported that printed aligners showed greater trueness and precision than thermoformed ones. However, the authors admitted that the study’s findings might have been affected by limitations encompassing the sample size calculation and the method of scanning the intaglio surface of the aligners. The authors also advocated the in vivo validation of the collected in vitro data [35].

Concerning the mechanical properties, based on the consideration that the polymerization of the printed devices continues for a certain time after printing, Milovanovic et al. [36] evaluated the effect of the storing conditions on clinically relevant mechanical properties, such as tensile, compressive, flexural strength, and strain at failure of the Dental LT Clear V1 resin (Formlabs Inc.). The results indicated that the resistance to tensile, compressive, and bending loads increased with time while the elongation at failure was reduced. The best performance of the aligner could be achieved 7 days after production [36].

Jindal et al. [37] compared the resistance to a compressive load mimicking bite force pattern of Duran or Durasoft thermoformed aligners (Scheu Dental, Iserlohn, Germany) with that of aligners 3D printed in Dental LT resin (Formlabs Inc.). The finite element analyses indicated a comparable behavior of the materials in terms of von Mises stress distribution [37]. However, the same research group had previously reported that Dental LT resin aligners had a maximum load resistance higher than Duran aligners [28]. In addition, reversible elastic deformation of Dental LT resin aligners was considered more favorable compared to irreversible plastic deformation of Duran aligners [28]. 

Clear aligners 3D printed in Tera Harz TC-85 (Graphy Inc.) were reported by Lee et al. to have higher flexibility and elastic range than aligners thermoformed from PET-G (Easy-Vac Gasket, 3A MEDES, Goyang-si, Republic of Korea), and were considered to allow a greater amount of tooth movement per aligner [29]. In addition, Tera Harz TC-85 resin (Graphy Inc.) proved to be geometrically stable at high temperatures and able to recover its original shape after deformation [29]. It should be noted that the reading of this paper was hampered by the misplacement of the Materials and Methods section at the end of the article.

Dental aligners 3D printed with DLP technology (Asiga Max, SCHEU-DENTAL, GmbH, Iserlohn, Germany) in shape memory polymer ClearX v.1.1 (Kline-Europe, Dusseldorf, Germany) were applied to a typodont and reported to produce biocompatible orthodontic forces regardless of the aligner thickness (0.8 mm and 1 mm). They were also considered capable of moving teeth after suitable thermal stimulus [38]. However, the authors themselves admit that the study had many limitations. The experimental model of simulating orthodontic movement and recording the forces delivered by the aligners was indeed far from reflecting the clinical situation. Moreover, it was not reported whether the protocol followed in the 3-point bending test was in accordance with international standards.

Zinelis et al. [39] demonstrated that the mechanical properties of the Tera Harz TC-85 resin were significantly affected by the printing device [39]. Particularly, aligners printed with liquid crystal display (LCD) technology had higher hardness, indentation modulus, and elastic index than those produced by DLP. Printed aligners were found to have hardness similar to thermoformed PET-G polymer and lower than Invisalign. It was thus implied that printed aligners would be more susceptible to wear than Invisalign. The modulus of elasticity of the 3D-printed resin was higher than that of thermoformed PET-G and comparable to Invisalign. 

Dental LT resin (Form labs Inc., Somerville, MA, USA) was tested after different post-curing protocols [40]. Post-cured aligners exhibited higher resistance to compressive strength than uncured ones, and higher post-curing temperatures proved to be more beneficial [40]. Still concerned with the post-print processing, Xu et al. reported that the flexural strength of Dental LT Clear Resin (Formlabs Inc.) was decreased by increasing the post-rinsing time with isopropyl alcohol (IPA) [41]. The study also showed that prolonged rinsing produced surface alterations that were not visible in samples post-rinsed for less than 1h. However, it should be noticed that the use of Pearson’s linear correlation test precluded the identification of the rinsing time interval over which the reduction in flexural strength had become statistically significant. It should also be pointed out that flexural strength was assessed with reference to the ISO 20795-2:2013 standard, which relates to ‘orthodontic polymers and copolymers used for the construction of active and passive orthodontic appliances’. Conversely, Milovanovic et al. [36] referred to ISO 178:2019, relating to ‘rigid and semi-rigid plastics’, for their 3-point bending test of the same resin. 

As IPA is flammable and somewhat toxic, alternative solutions were taken into consideration for rinsing. The several solutions investigated by Lambart et al. [42] had a similar effect to IPA on the roughness and cytotoxicity of the FREEPRINT^®^ splint 2.0 resin (Detax, Ettlingen, Germany). Only ethanol produced a significant reduction in flexural strength; however, the values measured in all the groups were deemed acceptable by the authors. The authors also stated that further evaluation is needed prior to using such solutions for appliances that fall in the category of medical devices [42]. 

Can et al. [43] aimed to assess the effect of in vivo aging on the mechanical properties of aligners printed in Tera Harz TC-85 resin (Graphy Inc.) and reported that the mechanical behavior of aligners retrieved after 1 week of clinical service did not significantly differ from that of unused appliances that had been printed under the same conditions. However, only six aligners coming from four patients were tested [43]. 

#### 3.2.2. 3D-Printed Fixed Retainers

Flexural strength, elastic properties, deflection, and creep of 3D-printed fixed orthodontic retainers of different thicknesses (0.8, 1, 1.2 mm) were investigated by Firlej et al. [44]. Retainers were printed with SLA Phrozen MINI4k printer (Phrozen Tech, Hsinchu City, Taiwan), using Nextdent MFC Crown and Bridge N1 resin (Vertex-Dental B.V., Soesterber, The Netherlands). The investigated properties depended on the thickness of the sample but were not directly proportional to it. Namely, the samples with the highest thickness (1.2 mm) had the highest values of modulus of elasticity and the lowest deformability under load, while the lowest modulus of elasticity and the highest deformability were recorded in 1 mm-thick specimens. 

#### 3.2.3. 3D-Printed Orthodontic Brackets

The opportunity offered by the 3D printing technologies to fully individualize size, shape, and prescription of the brackets is appealing [5,11,24,45].

Mechanical properties of 3D-printed orthodontic brackets made of Temporary CB resin (Formlabs Inc.) and Permanent Crown resin (Formlabs Inc.) were assessed [45]. After being subjected to instrumented indentation testing, both materials showed comparable Martens hardness, indentation modulus, and elastic index, and their mechanical characteristics were considered superior to those of commercially available plastic brackets [45]. The sliding resistance of a 3D-printed Self-ligating Shark SL bracket (Dentalline GmbH & Co. KG, Birkenfelt, Germany) was assessed in comparison to two ceramic, two metal, and one plastic commercially available bracket types, and in combination with archwires of different alloys (nickel–titanium, titanium–molibdenum, stainless steel) and cross-sections [46]. For all bracket-archwire combinations, 3D-printed polymer brackets showed a sliding resistance similar to that of commercially available polymer brackets and lower than that of ceramic and metal brackets [46].

#### 3.2.4. 3D-Printed Springs 

Othman et al. [47] used a flexible resin-based experimental material (Code: BM2008, GC, Tokyo, Japan) to 3D print springs of different coil diameters and lengths with a DLP printer. The amount of torque measured in vitro was more affected by coil diameter than by coil length. The same experimental material was used by Strobele et al. [48] to investigate the effect of coil spring height (4, 6, 8, 10, 12 mm) on compression resistance, and the greatest values were demonstrated by the shortest springs.

#### 3.2.5. 3D-Printed Palatal Plates

Among the potential applications of 3D printing digital technologies, there is the possibility of manufacturing palatal plates for newborns and infants with cleft palate (cleft covering plate), trisomy 21 (stimulating plate for Castillo Morales concept), or Robin Sequence (Tübingen palatal plate) [49,50,51]. 

Aretxabaleta et al. [49] used different materials to additively (AM) or subtractively (SM) manufacture samples of a Tübingen palatal plate for Robin Sequence treatment. V-Print splint (VOCO, Cuxhaven, Germany), FREEPRINT^®^ splint 2.0 (Detax), and FREEPRINT^®^ ortho (Detax) with DLP, Dental LT Clear V1 resin (Formlabs Inc.) with SLA, milled Yamahachi PMMA D4 (Yamahachi Dental MFG., Co., Gamagori, Japan), milled Smile PEEK (Pressing Dental, S.r.l., Falciano, Repubblica di San Marino), and cold-cure acrylic resin (Orthocryl clear, Dentaurum, Ispringen, Germany) were compared. Time efficiency and affordability of the materials, fracture load, and breaking pattern of the samples were measured to test safety and suitability. Dental LT Clear V1 resin was recommended, as it proved as a safe and affordable material [49]. The same research group performed another investigation to evaluate the trueness and precision of palatal plates manufactured using SLA (Dental LT Clear V1), DLP (V-Print splint), or SM (Yamahachi PMMA and Smile PEEK) in comparison with that of plates made in a conventional way from cold polymerizing resin (Orthocryl clear) [50]. All materials and technologies were considered adequate for clinical use. Superior trueness and precision were observed in SM plates in comparison to AM plates, but significant differences between the two SM materials were present, with Yamahachi PMMA performing significantly better. Among the AM technologies, DLP showed higher trueness and precision than SLA. All CAD/CAM manufactured plates had more favorable characteristics than conventionally manufactured ones, excluding the trueness of SLA-manufactured plates. Additionally, the trueness, accuracy, and manufacturing time of DLP printed plates significantly depended on the layer thickness. The authors concluded that the optimal layer thickness was 100 μm [50]. 

#### 3.2.6. 3D-Printed Metal Appliances

Zinelis et al. [10] assessed the elemental composition, mechanical properties, and electrochemical behavior of 12 molar distalizers 3D printed in a cobalt–chromium alloy after at least 7 months of intraoral use. Twenty alloy blocks printed under the same conditions served as controls. The printed devices had a highly homogeneous structure. The mechanical properties remained stable over the clinical service. However, intraoral aging degraded the superficial oxide layer, which has a protective effect against corrosion [10]. 

### 3.3. Biocompatibility

Biocompatibility is a clinically relevant property that the recent literature on 3D-printed aligners has taken into consideration [41,42,52]. According to the current EEC Directive 93/42 on medical devices, materials for long-term intra-oral use, i.e., a period longer than 30 days, must meet the biocompatibility requirements of Class IIa [53].

In the study by Xu et al. [41], Dental LT Clear Resin (Formlabs Inc.) did not demonstrate cytotoxic effects other than those of Orthocryl Clear acrylic resin (Dentaurum), tested as reference material, and of titanium, tested as a negative control. The authors deduced that post-polymerization procedures and rinsing with IPA for even just 5 min were sufficient to reduce the number of residual monomers below the cellular tolerance threshold [41]. 

Ahamed et al. [52] compared the cytotoxicity of the photopolymerizable resins Dental LT (Formlabs Inc.) and E-Guard clear (EnvisionTEC, Rockhill, SC, USA) with that of SmartTrack Invisalign (San Jose, CA, USA), a thermoformable material based on polyurethane. All three materials showed slight cytotoxicity measured with MTT assay on mice fibroblast cells (MFCs). E-Guard clear and Dental LT showed significantly lower levels of cell viability compared to SmartTrack Invisalign. The cytotoxicity of materials showed a gradual decrease with time [52]. 

Lambart et al. [42] reported that specimens of the 3D-printed FREEPRINT^®^ splint 2.0 resin (Detax) did not have cytotoxic effects when rinsed with IPA and other solutions tested in alternative.

In the abovementioned studies [41,42,52], the cytotoxicity tests were performed with reference to the ISO 10993-5 and 10993-12 standards relating to the cytotoxicity of medical devices.

Pratsinis et al. [54] evaluated the cytotoxicity and estrogenicity of aligners printed in the Tera Harz TC-85A resin (Graphy Inc.) with the SprintRay Pro 55 3D printer (SprintRay Inc.) and stored for 14 days in water at 37 °C. The eluates released by the resin did not adversely influence the viability of human gingival fibroblasts that had been exposed to them for 14 days. In addition, no xenoestrogenic activity was observed. Based on these findings, the authors concluded that the tested resin is biocompatible [54]. 

Still for Tera Harz TC-85A resin (Graphy Inc.) Willi et al. [55] assessed the degree of conversion and measured the amount of UDMA and BPA that leached from aligners stored in water at 37 °C for 1 week. The degree of conversion of the resin was found to be 83%, and no leaching of BPA was detected. Nevertheless, varying amounts of UDMA monomer were released by the stored specimens and may represent a health hazard that deserves further investigation [55]. 

It should indeed be considered that the aging method adopted by Pratsinis et al. [54] and Willi et al. [55], only involving aqueous immersion of the aligner, underestimated the effect on resin degradation and leaching of the factors acting intraorally, such as pH, temperature, bacterial and enzymatic activity, occlusal and masticatory forces [56,57].

Nakano et al. [56] developed a 3D-printable biocompatible resin composed of low-toxicity water-soluble monomers alone (Okamoto Chemicals Resin, 3D-1M, Patent 6042523) and subjected it to cytotoxicity (LDH test), proliferation (WST1 test) and mechanical testing. The results showed low cytotoxicity of the investigated material, but the composition ratio of raw materials affected cell survival. In addition, direct aligners produced via DLP using this resin lacked optimal mechanical properties, and further improvements are required [56]. 

### 3.4. Aesthetic Properties

The literature is scarce regarding the aesthetic properties of 3D-printed orthodontic devices for intraoral use. Only two articles on this topic were retrieved [11,34].

#### 3.4.1. 3D-Printed Retainers

Martorelli et al. [34] evaluated the patient’s aesthetic perception of 3D-printed and CNC-milled aligners. Patients’ preference was toward the milled aligners. The authors considered the so-called ‘staircase’ effect of AM as a possible explanation for the less satisfactory aesthetics of 3D-printed aligners. 

#### 3.4.2. 3D-Printed Brackets

Haynie et al. [11] investigated the color stability of brackets 3D printed with Dental LT, Dental SG, and Clear Resins (Formlabs Inc.). The brackets were immersed in different coloring solutions and exposed to accelerated aging. Color and translucency measurements were performed with a spectrophotometer before and after exposure to different conditions. All three materials showed pronounced changes in color when exposed to staining agents. Dental SG and Clear Resin also showed changes in color with aging; only the Dental LT resin demonstrated satisfactory color stability. The authors concluded that the color variations observed in the tested resins did not support their clinical use [11].

## 4. Discussion

The review of the literature revealed that aligners and retainers represent the currently most studied 3D-printed orthodontic appliances. Claimed advantages of 3D-printed aligners in comparison with thermoformed ones are greater accuracy and shortening of the supply chain, and reduction in costs, as the production of physical models can be avoided [25]. Moreover, the thermoforming procedure has been reported to adversely affect the mechanical and esthetic properties of the aligners [25]. In addition, 3D printing produces less waste, thus presenting as a more sustainable process. Additionally, 3D printing theoretically enables the production of aligners with customized thickness and the control of thickness across the arch, thus influencing the mechanical properties of the aligners [25]. 

Several investigations have been performed to evaluate the accuracy and the mechanical behavior of aligners materials. However, the collected evidence remains quantitatively scarce and of limited reliability. A large variability emerged in the research protocols, probably as a reflection of the current lack of universally agreed reference standards for in vitro testing of aligners materials, particularly the 3D printable ones, which are still relatively few and new.

Concerning mechanical properties testing, in some studies, instrumented indentation testing was used according to ISO 145577-1 to measure Marten’s Hardness, Indentation modulus, and Elastic index of 3D-printed resins and alloys [10,43,45]. 

In other investigations, the 3-point bending test was utilized to assess the flexural strength of 3D-printed resins, but differences among the experimental set-ups emerged in the specimens’ dimensions [29,42,44] and design (beam or dumbbell shape) [29,42,44], as well as in the ISO specification taken as a reference [36,41]. Either ISO 20795-2:2013 for orthodontic polymers and copolymers or ISO 178-4:2019 for plastics was considered. 

Moreover, the concern has been raised that the anisotropicity of AM aligners may affect their mechanical behavior, with print orientation being a possibly influential factor. However, this variable has so far been investigated only for its effect on the accuracy of the prints [30] rather than on their mechanical properties. As a matter of fact, standards for mechanical testing of 3D-printed resins, properly addressing the issues of print direction and sample size, have not been provided yet. Regarding sample size, in consideration of the structural variability of AM materials, the five specimens requested by ISO 20795-2:2013 and ISO 178-4:2019 may be too few to properly evaluate 3D-printed polymers.

It is also worth mentioning that stress relaxation, a property regarded as clinically relevant for thermoformed aligners to predict force decay with the use [29,38,58], has been assessed only for Tera Harz TC-85 [29] and ClearX v.1.1 [38].

In the majority of the reviewed studies, aligners and retainers were printed using SLA 3D printing technology. The accuracy of SLA 3D-printed aligners was superior to that of thermoformed aligners [28]. However, when different printing technologies were compared to each other, SLA and PPP were the most accurate, while DLP and cDPL were the most precise [26]. Unfortunately, the authors did not disclose the specific materials that were used for printing; they only reported that the materials were recommended by the manufacturer of each printer. It was also reported that both SLA [23] and DLP [25] printers produce thicker aligners compared to the digital design.

The most investigated 3D-printable material for the production of aligners and retainers is Dental LT Clear Resin (Formlabs Inc.). It was shown that, compared to traditional thermoformed aligners, 3D-printed Dental LT Clear Resin aligners had greater accuracy [28], similar resistance to compressive loads mimicking bite force patterns [37], and higher maximum load resistance due to reversible elastic deformation [28]. Milanovic et al. advised delivering aligners printed with Dental LT Clear V1 resin to the patient 7 days after production since the best mechanical properties were achieved after that time [36]. The accuracy of 3D-printed aligners made of Dental LT Clear Resin was not influenced by printing orientation nor by the duration of post-print UV treatment [26]. The fit of aligners 3D printed with Dental LT Clear Resin was inferior to that of traditionally vacuum-formed and thermoformed retainers. Nevertheless, the adaptation was still rated as acceptable for clinical use [33]. 

The cytotoxicity of Dental LT Clear Resin, though greater than that of thermoformed materials [52], was comparable with that of acrylic resin [41]. According to Xu et al. [41], since the removal of cytotoxic methacrylate monomers can be achieved by post-rinsing in IPA in 5 min, a further extension of this procedure does not increase the cytocompatibility of the resin, while it may result in a decrease in its flexural strength. 

Still concerning the Dental LT Clear Resin, it was pointed out that the material, although deemed as suitable for printing aligners, is not intended for this application but rather to print occlusal splints, orthodontic retainers, and other rigid orthodontic appliances [3,25,59]. 

The recently introduced photopolymer for direct printing of aligners Tera Harz TC-85 is claimed to have shape memory ability [25,29]. Lee et al. [43] stated that the flexibility, elasticity, and thermostability of this material could enhance the efficiency of the aligners [29]. Other studies reported that the mechanical behavior of this resin was acceptable [39] and was not altered by intraoral aging. In addition, a high degree of monomer conversion, as well as a lack of cytotoxicity and estrogenicity, were verified for this material [54,55]. 

In general, the evidence so far collected on cytotoxicity and estrogenicity of 3D-printed resins for aligners, retainers, and occlusal splints is reassuring [41,42,52,53,54,55,56]. 

It should, however, be mentioned that all the biocompatibility tests were based on substances released from the specimens by passive hydrolysis and failed to assess the effects of intraoral influential factors, such as salivary enzymes, functional and parafunctional loads, temperature variations, pH changes such as those related to the consumption of soft drinks or juices [57], microbial activity. Such experiments omit to evaluate the challenge of the oral environment on polymers degradation and are only useful to compare 3D-printed appliances with other commercially available or conventionally manufactured devices.

It is also worth reporting that in order to reduce the cytotoxicity and allergic potential of 3D-printed resins, methods to coat these materials with highly biocompatible, natural polymers, such as chitosan, are being explored [60].

The aesthetic properties of 3D-printable materials for use in orthodontics, despite the obvious clinical relevance, are almost completely unexplored. One study on the aesthetic appearance of 3D-printed brackets suggested that satisfactory color stability could not be achieved [11]. Further improvements of 3D printable materials appear necessary to gain aesthetic properties that are adequate for orthodontic devices such as brackets or aligners. 

Only two studies investigated the effect of aging on the properties of 3D-printed materials. Can et al. [43] assessed the mechanical and electrochemical characteristics of aligners specimens after 1 week of intraoral use, while Zinelis et al. [10] did the same evaluation on metallic distalizing appliances retrieved after at least 7 months of clinical service.

Concerning with in vivo studies, the clinical evidence so far collected on 3D-printed orthodontic appliances is limited to case reports and proof of concept cases.

None of the studies involved a longitudinal evaluation of the clinical outcome of the additively manufactured devices. The assessment of time-dependent properties of materials is clinically relevant also for aligners, despite the relatively short intraoral stay of each set, which is typically 2 weeks. The stress relaxation phenomenon and the viscoelastic behavior that was reported to be crucial for the clinical efficacy of thermoformed aligners [25,58] should be properly investigated also for 3D-printed aligners. Adequately long follow-up data should be provided for 3D-printed retainers, brackets, and metallic appliances.

With regard to metal printed devices, it emerged from this literature review that few investigations focused on the properties of 3D-printed alloys, although the digital workflow for their production has been described in the literature [7,61,62,63,64], and considered as a simplification compared to the analog procedures [61]. In addition to avoiding patient discomfort with conventional impression-taking techniques and eliminating the need for a physical model, with 3D-printed metallic appliances, the step of teeth separation can also be skipped, as the structures do not extend in interdental spaces [61]. The versatility of digital design, allowing the production of multitasking appliances, the possibility to create a digital library, streamlining the design process, the remote communication with the laboratory or other practitioners, and widening the opportunities for cooperation have been indicated as advantages of 3D-printing metal appliances [61]. Among the disadvantages of the technology, the restricted choice of available alloys, their rigidity, and the current inability to print multiple alloys have been mentioned [61]. The stiffness of the alloys makes any minor mistake in the design and manufacture of the appliances quite unforgiving and makes rather difficult any chairside or intraoral adjustment through wire bending [61]. Additionally, the removal can be difficult with conventional band-removing tools, as 3D-printed devices typically exhibit good fit and smooth structures. In order to prevent such practical inconvenience and the accompanying risk of enamel fractures, debonding buttons or spikes, as well as weak points, can be introduced in the design of the appliances [61]. 

Despite the clinical relevance of the peculiar elastic behavior of 3D-printed alloys, only one study has been directed at assessing it [10].

No specific information is currently available on the biocompatibility of 3D-printed alloys used to manufacture orthodontic appliances.

These areas of research remain open to further laboratory and clinical investigations.

The present summary of evidence was denoted as a critical review since it implied an extensive search of the literature along with a critical analysis of its quality. Nevertheless, a formal quality assessment, such as a risk of bias assessment, could not be provided, since this type of analysis was not applicable to the current evidence, which is still scarce, heterogeneous, and almost completely missing clinical data. Still, the conceptual analysis presented in this review has the value of embodying the current evidence on the hot topic of 3D printing in orthodontics and highlighting the future needs for research in this largely expanding and rapidly progressing field. 

## 5. Conclusions

Current scientific evidence on 3D-printable materials for intraoral use in orthodontics is still quantitatively and qualitatively limited. It is expected that 3D printing technology will experience widespread use in everyday clinical practice in the very near future. Therefore, the scientific evidence should be significantly consolidated, both through the definition of standards for laboratory testing to be shared by the international scientific community, and by starting the necessary clinical investigations, according to rigorous and reliable protocols.

## Figures and Tables

**Table 1:** Search terms, search strategy, and number of documents retrieved.

MEDLINE/PubMed
orthodontics AND (3D printing OR additive manufacturing OR rapid prototyping)
(“orthodontal”[All Fields] OR “orthodontic”[All Fields] OR “orthodontical”[All Fields] OR “orthodontically”[All Fields] OR “orthodontics”[MeSH Terms] OR “orthodontics”[All Fields]) AND (“printing, three dimensional”[MeSH Terms] OR (“printing”[All Fields] AND “three dimensional”[All Fields]) OR “three-dimensional printing”[All Fields] OR (“3d”[All Fields] AND “printing”[All Fields]) OR “3d printing”[All Fields] OR ((“rapid”[All Fields] OR “rapidities”[All Fields] OR “rapidity”[All Fields] OR “rapidness”[All Fields]) AND (“prototypal”[All Fields] OR “prototype”[All Fields] OR “prototype s”[All Fields] OR “prototyped”[All Fields] OR “prototypes”[All Fields] OR “prototypic”[All Fields] OR “prototypical”[All Fields] OR “prototypicality”[All Fields] OR “prototypically”[All Fields] OR “prototyping”[All Fields])) OR (“addit manuf”[Journal] OR (“additive”[All Fields] AND “manufacturing”[All Fields]) OR “additive manufacturing”[All Fields]))
464 items
Scopus
orthodontics AND (3d printing) OR (additive manufacturing) OR (rapid prototyping)
( TITLE-ABS-KEY ( orthodontics ) AND TITLE-ABS-KEY ( 3d AND printing ) OR TITLE-ABS-KEY ( additive AND manufacturing ) OR TITLE-ABS-KEY ( rapid AND prototyping ) )
205 documents
Cochrane Library
orthodontics AND 3d printing OR additive manufacturing OR rapid prototyping
orthodontics AND (3D printing OR additive manufacturing OR rapid prototyping) in Title Abstract Keyword
0 Cochrane reviews

## Data Availability

Not applicable.
